# Commonalities in Metabolic Reprogramming between Tobacco Use and Oral Cancer

**DOI:** 10.3390/ijerph191610261

**Published:** 2022-08-18

**Authors:** Blake R. Rushing, Spencer Tilley, Sabrina Molina, Madison Schroder, Susan Sumner

**Affiliations:** 1 Department of Nutrition, University of North Carolina at Chapel Hill, Durham, NC 27599, USA; 2 Nutrition Research Institute, University of North Carolina at Chapel Hill, Kannapolis, NC 28081, USA

**Keywords:** metabolomics, metabolism, oral cancer, smoking, tobacco use, carcinogen, cancer, metabolic reprogramming

## Abstract

Tobacco use is a major public health concern and is linked to myriad diseases, including cancer. The link between tobacco use and oral cancer, specifically, is very strong, making tobacco use one of the primary risk factors for oral cancer. While this association is well known, the underlying biochemical changes that result from tobacco use, and how this links to metabolic phenotypes of oral cancer, is not well understood. To address this knowledge gap, a combination of literature reviews and metabolomics studies were performed to identify commonalities in metabolic perturbations between tobacco use and oral cancers. Metabolomics analysis was performed on pooled reference urine from smokers and non-smokers, healthy and malignant oral tissues, and cultured oral cells with or without treatment of the well-known tobacco carcinogen 4-(methylnitrosamino)-1-(3-pyridyl)-1-butanone (NNK). Alterations in amino acid metabolism, carbohydrates/oxidative phosphorylation, fatty acid oxidation, nucleotide metabolism, steroid metabolism, and vitamin metabolism were found to be shared between tobacco use and oral cancer. These results support the conclusion that tobacco use metabolically reprograms oral cells to support malignant transformation through these pathways. These metabolic reprogramming events may be potential targets to prevent or treat oral cancers that arise from tobacco use.

## 1. Introduction

Tobacco use remains a leading cause of disease and mortality both in the United States and worldwide. There are an estimated 1.3 billion tobacco users worldwide, and that figure is projected to reach 1.6 billion in the next 25 years [1]. Globally, over 7 million deaths each year are caused by tobacco use, resulting in an ongoing public health crisis [2]. Tobacco products are used in many forms, such as cigarettes, chewing tobacco, cigars, and hookahs. Smoking tobacco and exposure to secondhand smoke are known to be major causes of cancer, and cigarette smokers have significantly higher odds ratios for developing oral cancer compared to non-smokers [3]. Despite advances in the understanding of oral cancer occurrence and treatment, survival rates remain low, indicating a need to better understand methods for preventing and therapeutically targeting oral cancers, particularly those that arise from tobacco use.

Metabolic reprogramming is a hallmark of cancer, with multiple metabolic adaptations occurring in cells and tissues during the initiation, promotion, and progression of cancers. These metabolic alterations allow cancers to sustain increased proliferation, increase defenses against oxidative stress, evade surveillance by the immune system, and perform many other processes that give growth and/or survival advantages [4,5,6,7]. Targeting these metabolic reprogramming events has become a popular strategy for the prevention and treatment of cancers, as demonstrated by the FDA-approved chemotherapeutics that target cellular metabolism, such as methotrexate [8].

Tobacco and tobacco smoke contain hundreds to thousands of chemical constituents that can potentially influence cellular metabolism. Among the most well studied are the different types of carcinogens contained within tobacco. Most of these carcinogens can be categorized into three main chemical classes: nitrosamines, benzopyrenes, and aromatic amines [9]. When tissues are exposed to carcinogens or other toxic substances, metabolic changes occur in response to cellular/tissue/genetic damage. These metabolic changes may facilitate the early stages of cancer development and may persist into the later stages of cancer [10]. Knowing the specific types of metabolic perturbations that occur due to tobacco use, and whether or not they persist in oral cancer tissues, may inform new intervention strategies to prevent oral cancer occurrence or improve oral cancer treatment, particularly in tobacco users. The goal of this project was to understand the differences in metabolomes in oral cancer tissue compared with healthy oral tissues, and to determine if these metabolic perturbations are also present in tobacco smokers compared with non-smokers. To investigate this, literature reviews and high-performance liquid chromatography high resolution mass spectrometry (HPLC-HR-MS) metabolomics analyses of human biospecimens/cultured cells were performed to identify common metabolic perturbations between tobacco users and individuals with oral cancer. This new information can be used to better understand how tobacco usage leads to oral cancer development and to potentially uncover metabolic pathways or metabolites that can be targeted to treat or prevent oral cancers.

## 2. Materials and Methods

### 2.1. Literature Review 

#### Searches and Study Selection

Two literature reviews were performed using PubMed, UNC Library’s “articles+” database, and Google Scholar search engines. The first literature review searched for observational studies, cell studies, and animal studies concerning tobacco products and changes in metabolism using the search terms (“Smoking” or “Tobacco”) and (“Metabolism” or “Metabolites”). Studies were not included if the titles and/or abstract did not clearly describe the impact of smoking on metabolism. In the second literature review, observational studies concerning changes in metabolism and oral cancer status were found using the search terms “Oral Cancer” and (“Metabolism” or “Metabolites” or “Metabolomics”). Studies were removed if they were determined to be irrelevant based on either title or abstract, and whether or not metabolism changes and oral cancer status were reported. Studies were included independent of study design. Studies were included in the first literature review if they investigated the relationship between metabolites and smoking. All studies focused on smoking cessation were excluded. In the second literature review, studies were included if they looked at oral cancer and associated metabolic changes. Studies that focused on other forms of cancer were excluded. Studies were not excluded from either literature review based on population size. Studies were also not excluded if the population had an abnormal hormonal state such as pregnancy. Studies only investigating exogenous metabolites without a focus on host metabolism were excluded. A broad array of metabolites of host metabolism was examined for both reviews.

### 2.2. Preparation of NIST Reference Urine from Smokers and Non-Smokers for UHPLC-HR-MS Metabolomics

Pooled urine reference materials from smokers and non-smokers were purchased from the National Institute of Standards and Technology (NIST). Ten 50 μL aliquots each of NIST 3672 (smokers’ urine) and NIST 3673 (non-smokers’ urine) were placed into individual tubes (*n* = 10 per group). Quality Control Study Pools (QCSPs) were made by combining an additional 300 μL from both reference materials and aliquoting the mixture at a volume of 50 μL. Additional 50 μL aliquots of LC-MS grade water were prepared for blank samples. Each sample was extracted by the addition of 400 μL of methanol containing an internal standard (500 ng/mL tryptophan-d5). Samples were vortexed for 2 min at 5000 rpm, and then centrifuged at 4 °C for 10 min at 16,000 rcf. The remaining supernatant, 350 μL, was dried under speedvac, reconstituted in 100 μL of a 5% methanol solution, and transferred to LC-MS vials. The individual smoker and non-smoker samples were randomized and analyzed by UHPLC-HR-MS with blank and QCSP injections inserted at a rate of 10%. 

### 2.3. Preparation of Malignant and Non-Malignant Oral Tissue from a Tissue Microarray (TMA) for UHPLC-HR-MS Metabolomics

Healthy (*n* = 10) and cancerous (*n* = 50) oral tissue samples were purchased from US Biomax, Inc. (Derwood, MD, USA) in the form of a tissue microarray (OR601c, fixed-formalin paraffin wax format), which was stored at 4 °C. Metabolites from tissues were extracted by first placing 2 µL of an extraction solution (80:20 methanol:water) onto each tissue section. Using a pipette tip, tissue sections were minced in the 2 µL droplets and were then transferred by pipette to a tube containing an additional 50 µL of extraction solution. Another 2 µL droplet was placed onto the location of the tissue section and then the process was repeated to recover any remaining tissue. Blanks were prepared by performing this process on an empty location of the TMA slide that did not contain tissue but had paraffin wax. Samples were vortexed for ten minutes at 5000 rpm and then centrifuged for ten minutes at 16,000 rcf at 4 °C. Afterwards, 40 μL of the supernatants were transferred into LC-MS vials. A QCSP was made by mixing 5 μL from each individual study sample into one LC-MS vial. The individual study samples were then randomized and analyzed by UHPLC-HR-MS with blank and QCSP injections inserted at a rate of 10%. 

### 2.4. Preparation of CAL-27 Cells Treated with Vehicle or the Tobacco Carcinogen 4-(Methylnitrosamino)-1-(3-pyridyl)-1-butanone (NNK) for UHPLC-HR-MS Metabolomics

CAL-27 cells were purchased from the American Type Culture Collection (ATCC) and were cultured according to manufacturer protocols. Cells were cultured in Dulbecco’s Modified Eagle Medium (DMEM) supplemented with 10% fetal bovine serum (FBS), 2 mM glutamine, and 1% penicillin-streptomycin. Prior to treatment, cells were seeded into 12 well tissue culture plates to achieve 70–80% confluency (~0.4 × 10^6^). Cells were treated (*n* = 3 per group) with 100 µM NNK or vehicle (0.1% DMSO). The cells were then incubated for 24 h and then the metabolites of the cells were harvested as described previously [11]. Briefly, media was aspirated and cells were quickly washed with ice-cold PBS to remove residual media. After aspirating PBS, 1 mL of ice-cold 80% methanol was added to cells to quench metabolism. Cells were removed from plates by scraping and were lysed by three freeze–thaw cycles. An additional 80% methanol was added to cell extracts to normalize by protein content. Extracts were centrifuged for ten minutes at 16,000 rcf at 4 °C, and supernatants were transferred to LC-MS vials for analysis. A QCSP was made by combining 10 μL from each individual study sample into one LC-MS vial. The individual study samples were then randomized and run through LC-MS with blank and QCSP injections inserted at a rate of 10%.

### 2.5. Metabolomics Analysis via Ultra High-Pressure Liquid Chromatography Combined with High Resolution Mass Spectrometry (UHPLC-HR-MS)

Chromatographic and HRMS data were acquired on a Vanquish UHPLC system coupled to a Q Exactive HF-X Hybrid Quadrupole-Orbitrap Mass Spectrometer (Thermo Fisher Scientific, San Jose, CA, USA) using conditions according to published untargeted metabolomics methods [11,12,13,14,15,16]. Chromatographic data were acquired using an HSS T3 C18 column (2.1 × 100 mm, 1.7 µm, Waters Corporation, Milford, MA, USA) at 50 °C with binary mobile phases of water (A) and methanol (B), each containing 0.1% formic acid (*v*/*v*). The linear gradient consisted of an initial composition of 2% B, increased to 100% B over 16 min, and was held at 100% B for 4 min, with a flow rate at 0.4 mL/min. Data-dependent acquisition was used to acquire spectral data from 70 to 1050 *m*/*z*. The untargeted data were then processed using Progenesis QI (Waters Corporation). Data were filtered by removing peaks with a higher average abundance in blank injections as compared to QCSP injections. Peaks were normalized using the “Normalize to All” function in Progenesis except for the TMA samples, which were normalized to the total intensity. 

### 2.6. Metabolite Identification/Annotation

Peaks detected by UHPLC-HR-MS were identified or annotated by matching to an in-house reference standard RT, Mass, MS/MS library of over 2400 compounds run on the untargeted platform, or to public databases (NIST, METLIN, HMDB). To report the evidence basis for each metabolite match, an ontology system is given, based on matches by accurate mass (MS, <5 ppm), retention time (RT, ±0.5 min), and fragmentation similarity (MS/MS, >30). OL1 refers to an in-house library match by MS, MS/MS, and RT; OL2a refers to an in-house library match by MS and RT; OL2b refers to an in-house library match by MS and MS/MS; PDa refers to a public database match by MS and experimental MS/MS (NIST or METLIN); PDb refers to a public database match by MS and theoretical MS/MS (HMDB); PDc refers to a public database match by MS and isotopic similarity; PDd refers to a public database match by MS only.

### 2.7. Multivariate, Univariate, and Pathway Analysis of Metabolomics Data

**Figure 1:**
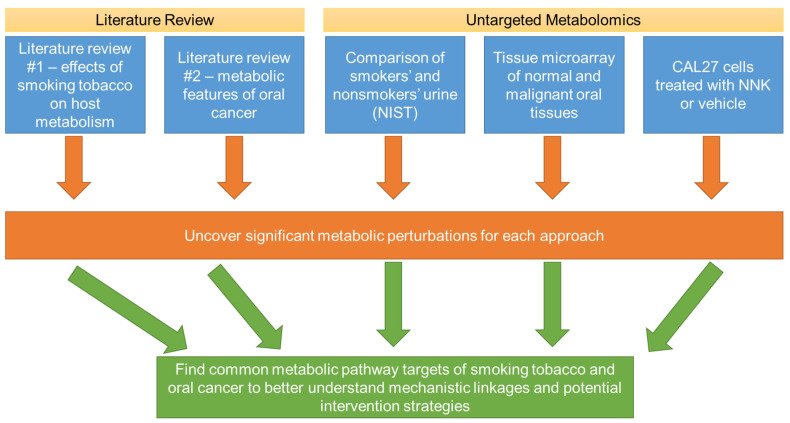
Overall workflow of the present study to identify commonalities of metabolic perturbations of smoking tobacco and oral cancer. Multiple strategies were utilized, including literature reviews and untargeted metabolomics investigations involving biospecimens for smokers and non-smokers, normal and malignant oral tissues, and an oral cancer cell line (CAL27) treated with a prominent tobacco carcinogen. Shared metabolic disruptions between these investigations are likely to reveal mechanistic insights to how smoking alters metabolism to lead to oral cancer, as well as potential metabolic targets to prevent or treat oral cancers that arise due to tobacco use.

A multivariate statistical analysis was performed on the preprocessed data using SIMCA 16.0.2 (Sartorius Stedim Data Analytics AB, Umeå, Sweden). Principle Component Analysis (PCA) models were created to ensure sufficient clustering of the QCSP samples (Figure 1), a QC benchmark commonly used in metabolomics studies [17]. Orthogonal partial least squares discriminate analysis (OPLS-DA) models were then created using the preprocessed data to evaluate group separations and calculate variable importance to projection (VIP) scores for each peak. Univariate statistics (*p*-value, fold change) were then calculated for each peak between study groups. The Exact Wilcoxon Rank Sum Test was used for all *p*-value calculations except for the CAL27 experiment, which used the Students’ *t*-test. Pathway analysis was performed using the “Functional Analysis” module in Metaboanalyst 5.0 [18]. Retention time, *m*/*z*, and calculated *p*-values were entered for each peak in each pairwise comparison. A mass accuracy of 5 ppm was used with all comparisons. Both the mummichog and GSEA algorithms were used for the pathway enrichment analysis. The default *p*-value cutoff in the mummichog algorithm that selects the top 10% of peaks was used for all analyses. A combined *p*-value from both GSEA and mummichog, calculated by Metaboanalyst, was used to identify significant pathways. *p*-values were not corrected for multiple comparisons.

## 3. Results

### 3.1. Literature Review

The first literature review on the impact of smoking on metabolism resulted in 24 articles that met the inclusion/exclusion criteria. The second literature review on the metabolic features of oral cancer resulted in 14 articles that met the inclusion/exclusion criteria. A list of host metabolites found to be altered between smokers compared to non-smokers, and between individuals with and without oral cancer can be found in Table 1 and Table 2, respectively. Overall, a greater number of metabolic pathways were found to be altered in the smoking vs. nonsmoking comparison compared to the oral cancer vs. noncancer comparison. Amino acids (particularly those involved in energy production and one-carbon metabolism), energy-producing metabolites (carbohydrates, fatty acids, lipids), antioxidant metabolites (e.g., glutathione) and some vitamin forms (particularly A and B vitamins) were the most prominent metabolic alterations found in the two literature reviews. 

### 3.2. Metabolomics Analysis of NIST Reference Material from Smokers and Non-Smokers

**Figure 2:**
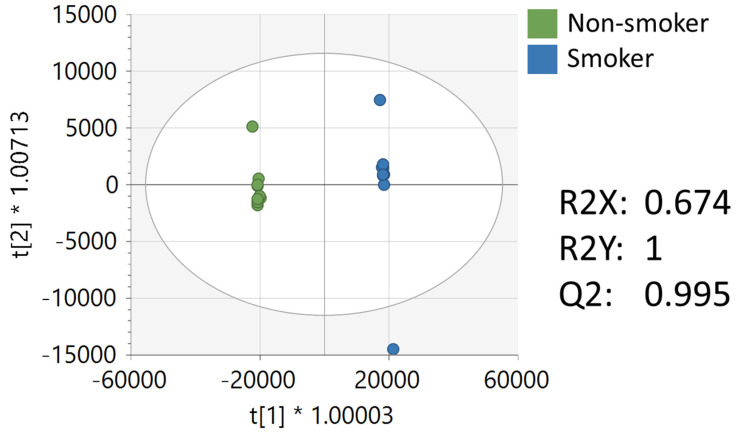
OPLS-DA of metabolomics data of NIST smoker and non-smoker urine reference materials.

**Figure 3:**
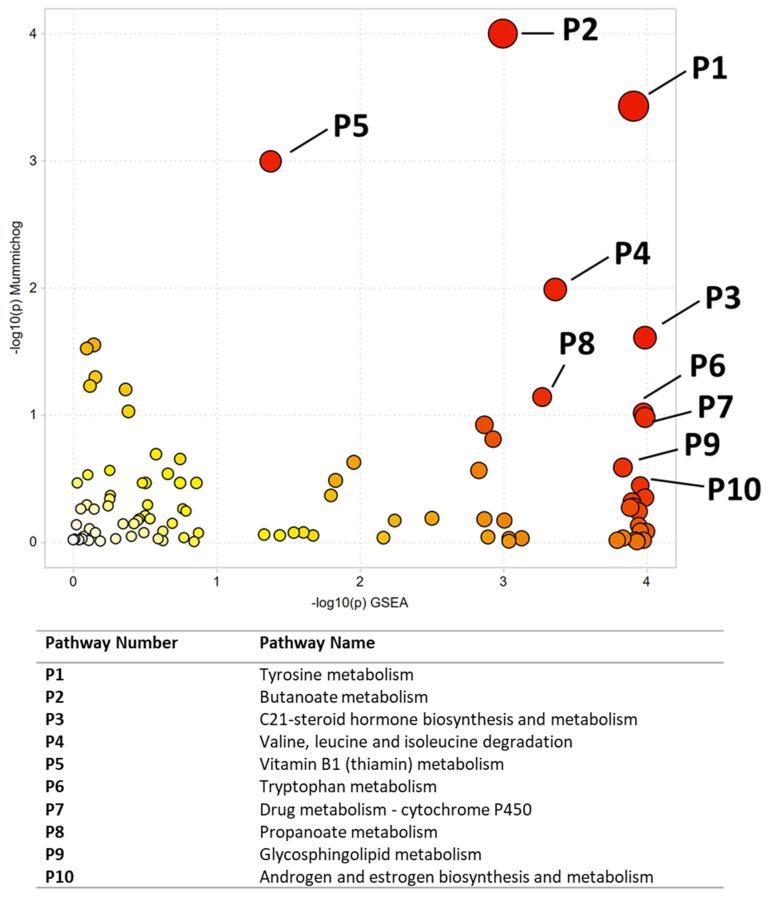
Pathway enrichment using Metaboanalyst for the comparison of NIST smoker and non-smoker urine reference material. All significant pathways are listed in Table 3. Darker circles represent pathways with a lower *p*-value. Larger circles indicate a higher number of significant metabolite hits relative to the total pathway size.

Multivariate, univariate, and pathway analysis was performed on the replicate injections from the NIST smokers’ reference urine (*n*= 10) and NIST non-smokers’ reference urine (*n*= 10). OPLS-DA (Figure 2) analysis was performed on all 16,136 peaks from the preprocessed data and showed strong model statistics for the differentiation of the two groups (R2X = 0.674, R2Y = 1), with good reproducibility (Q2 = 0.995). MetaboAnalyst was used to perform pathway analysis between the NIST smokers’ and non-smokers’ reference materials using the 16,136 peaks following data preprocessing. The plot of the −log10 (p) mummichog vs. −log10 (p) GSEA is shown in Figure 3, and significantly perturbed metabolic pathways (*p* < 0.05) are listed in Table 3. Table 4 lists the top 50 metabolites by *p*-value that differentiated the two groups that were matched at the OL1 ontology level. Additional significant metabolites (*p* < 0.05) that differentiated smokers and nonsmokers are listed in Supplementary Table S1. These compounds included amino acids (e.g., glutamine, phenylalanine), nucleotide metabolites (e.g., uracil, xanthine, adenosine, deoxyribose), and fatty acid oxidation intermediates (e.g., succinylcarnitine, isobutyryl-L-carnitine). As expected, several exogenous metabolites related to nicotine metabolism were found to be elevated in the NIST pooled smokers’ urine.

### 3.3. Metabolomics Analysis of Cancerous and Non-Cancerous Oral Tissue from a Tissue Microarray

**Figure 4:**
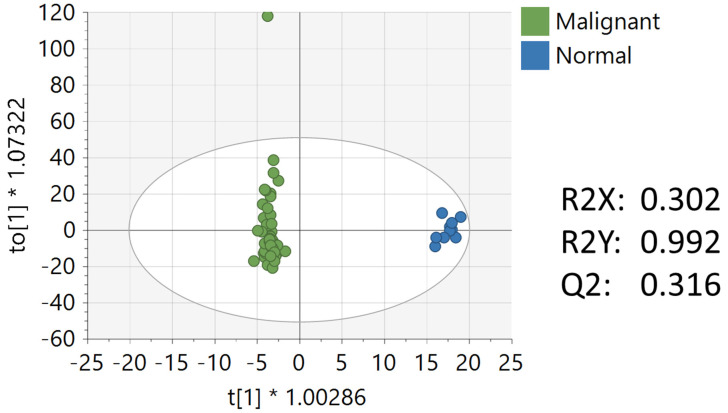
OPLS-DA of metabolomics data between malignant and normal oral tissues. Unit variance scaling was used for all peaks.

**Figure 5:**
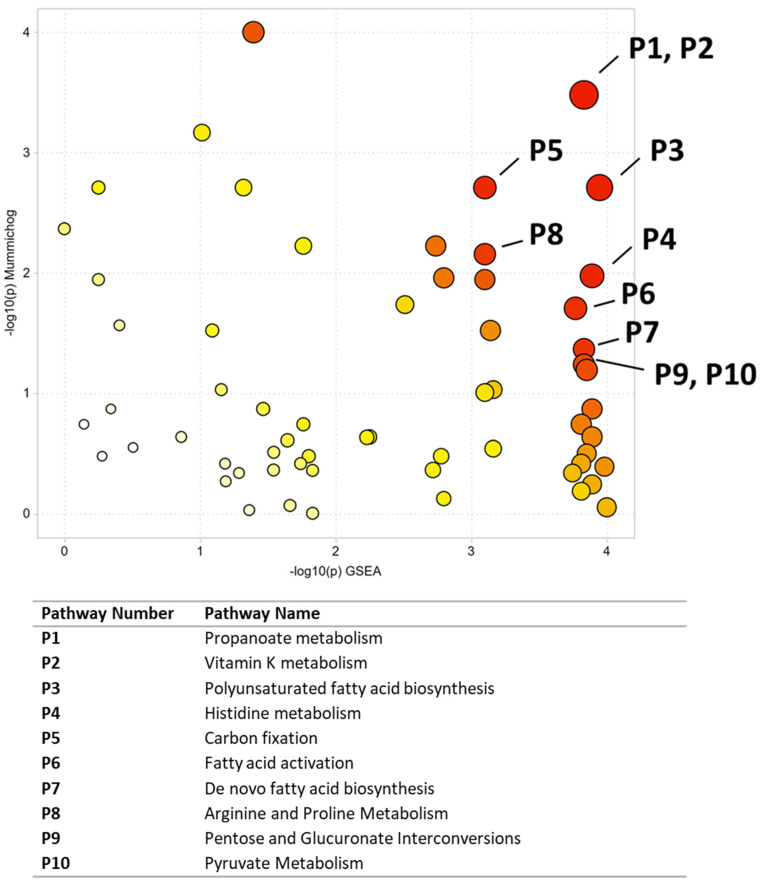
Pathway enrichment using Metaboanalyst to compare malignant and normal oral tissues. All significant pathways are listed in Table 5. Darker circles represent pathways with a lower *p*-value. Larger circles indicate a higher number of significant metabolite hits relative to the total pathway size.

Multivariate, univariate, and pathway analysis was performed on the malignant (*n* = 50) and normal (*n* = 10) tissue samples from the oral tissue microarray. OPLS-DA (Figure 4) analysis was performed on all 3490 peaks from the preprocessed data and showed strong model statistics for the separation between the two groups (R2X = 0.302, R2Y = 0.992), with good reproducibility (Q2 = 0.316). MetaboAnalyst was used to perform pathway analysis between the malignant and normal tissues using the 3490 preprocessed peaks. The plot of the mummichog −log10 (p) vs. the GSEA −log10 (p) is shown in Figure 5, and the top ten pathways by *p*-value are labeled and displayed in Table 5. Table 6 lists the top 50 metabolites by *p*-value that differentiated the two groups that were matched at an OL1, OL2a, OL2b, or PDa ontology level. These included nucleotide metabolites (e.g., 2-aminoadenosine, 5-methylcytosine, cytosine), intermediates of fatty acid oxidation (e.g., malonyl-carnitine), eicosanoid metabolites (e.g., eicosapentaenoate, 17,20-Dimethyl Prostaglandin F1α, 15(R)-15-Methylprostaglandin F2α, Leukotriene B3), and carbohydrate metabolites (e.g., galactose, glyceraldehyde). Of note, several exogenous metabolites related to plasticizers and pesticides were found to be significantly different between healthy and malignant oral tissues.

### 3.4. Metabolomics Analysis of CAL-27 Cells Treated with NNK

**Figure 6:**
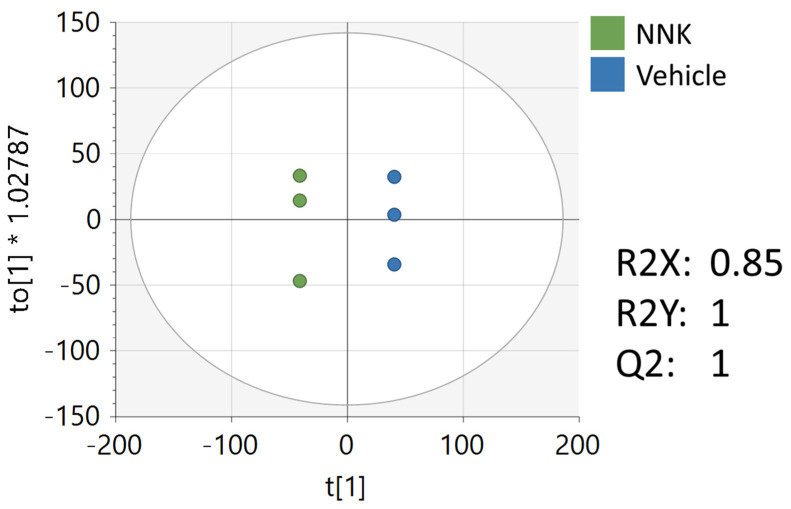
OPLS-DA of the metabolomics data from NNK and vehicle-treated CAL27 cells. Unit variance scaling was used for all peaks.

**Figure 7:**
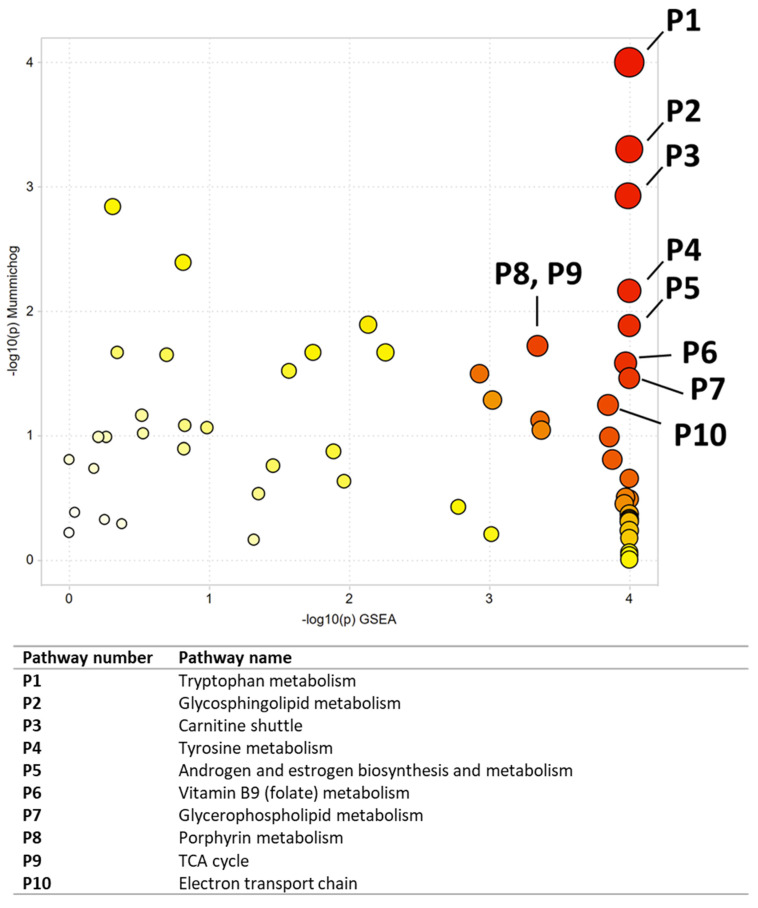
Pathway analysis results comparing NNK and vehicle-treated CAL27 cells. All significant pathways are listed in Table 7. Darker circles represent pathways with a lower *p*-value. Larger circles indicate a higher number of significant metabolite hits relative to the total pathway size.

Multivariate, univariate, and pathway analyses were performed on an oral cancer cell line (CAL27) treated with a NNK, a well-studied tobacco carcinogen, or vehicle (DMSO). OPLS-DA (Figure 6) analysis was performed on all 7225 peaks from the preprocessed data and showed strong separation between the two groups (R2X = 0.85, R2Y = 1) and good reproducibility (Q2 = 1). MetaboAnalyst was used to perform pathway analysis between NNK and vehicle-treated cells using the 7225 preprocessed peaks. The plot of the -log10 (p) mummichog vs. −log10 (p) GSEA is shown in Figure 7, and the top ten pathways by *p*-value are labeled and displayed in Table 7. Table 8 lists the top 50 metabolites by *p*-value that differentiated the two groups that were matched at an OL1, OL2a, OL2b, or PDa ontology level. Additional significant metabolites (*p* < 0.05) that differentiated smokers and nonsmokers are listed in Supplementary Table S2. These included intermediates of fatty acid oxidation (e.g., butanoylcarnitine, valerylcarnitine, propanoylcarnitine), amino acids (e.g., tyrosine, methionine, isoleucine, tryptophan), and TCA cycle metabolites (e.g., malic acid, pantothenate, NAD).

## 4. Discussion

### Commonalities between Altered Pathways between Studies

**Figure 8:**
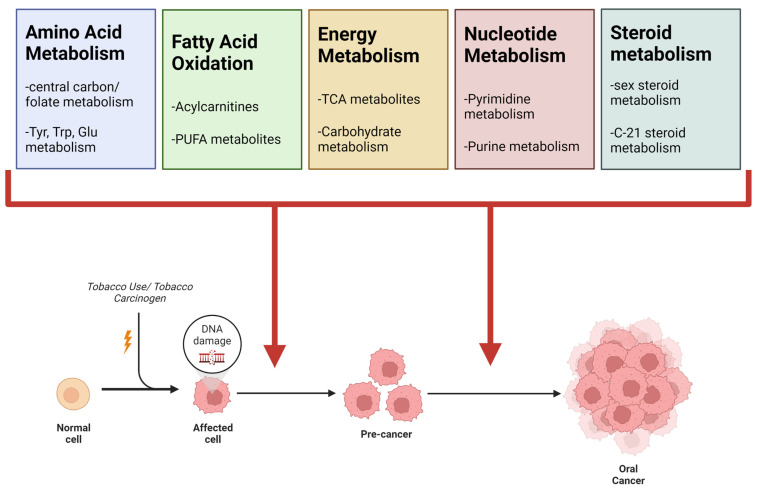
Summary of common metabolic alterations observed between tobacco use and oral cancer. From literature reviews and the original experiments described herein, four super pathways were found to be common between tobacco use and oral cancer. The most prominent sub-pathways are listed under each super pathway. Table 9 includes a more detailed view of pathways observed for each strategy.

*Common Alterations:* A summary of significant metabolic perturbations across all metabolomics studies and literature reviews is summarized in Table 9. Significant pathways were organized into super pathways, which are displayed if significant perturbations occurred in at least three of the five investigations. Amino acid metabolism, carbohydrate metabolism and oxidative phosphorylation, vitamin metabolism, fatty acid metabolism, polyunsaturated fatty acid (PUFA) metabolism, steroid metabolism, and nucleotide metabolism were the super pathways that met these criteria. Our investigations show that these metabolic processes are the major perturbations associated with smoking tobacco and oral cancer (Figure 8).

*Amino Acid Metabolism:* Perturbation in amino acid metabolism was reported in the reviewed literature and in all three metabolomics experiments described herein. Some amino acids that were important to the differentiation of smokers vs. non-smokers, NNK-treated vs. control cells, and in cancerous vs. noncancerous oral tissue included tyrosine, tryptophan, glutamine, and those involved in one-carbon metabolism (serine, glycine, methionine, etc.). Interestingly, there has been an increased interest in amino acid metabolism in cancer in recent years. In addition to their role in the biosynthesis of proteins to sustain increased cellular proliferation, amino acids and their metabolites have been shown to play a role in various other processes to promote oncogenesis including epigenetic regulation and modulation of immune function to support tumor growth and metastasis [57]. Furthermore, amino acids can act as alternate fuel sources within the cell, and can also support redox pathways and nucleotide synthesis to promote tumor growth and survival [57]. Our investigations found perturbations in amino acid metabolism in all experiments and in the literature reviews, supporting that oral cancers dysregulate amino acid metabolism and that smoking tobacco may predisposition oral cells to transition to malignancy by affecting these pathways. 

*Carbohydrate Metabolism and Oxidative Phosphorylation:* Perturbations in carbohydrate metabolism and oxidative phosphorylation was observed in all metabolomics studies and literature reviews. Dysregulations in carbohydrate metabolism is a well-known feature of many cancers, with the Warburg effect being one of the earliest properties of cancers known to the research community [58]. Many cancers have been shown to have an increase uptake of glucose, which corresponds to increased rates of glycolysis and lactate production [59]. In doing so, cancer cells are able to increase glycolytic precursors that allow for anabolic reactions to aid in sustaining increased proliferation. The TCA cycle and oxidative phosphorylation are also impacted, typically showing an increase in anabolic pathways, such as shunting citrate into lipid synthesis, rather than catabolic pathways to produce ATP [59]. Our results support the conclusion that perturbations in carbohydrate metabolism and oxidative phosphorylation are seen in oral cancer tissues and in biospecimens from tobacco smokers, suggesting that these metabolic processes are major targets by which smoking tobacco leads to oral cancer development.

*Fatty acid metabolism:* Fatty acid metabolism was altered in both literature reviews and all three experiments. Cancer cells have been shown to alter fatty acid metabolism to meet energy demands, synthesis membrane components to support proliferation, promote metastasis, and remodel the tumor microenvironment [60]. This signifies a potential role for fatty acid metabolism in not only establishing a tumor, but in facilitating the progression into more advanced stages. Several agents targeting fatty acid/lipid metabolism are in preclinical or clinical stages, and may have potential utility in treating oral cancers [60].

*Polyunsaturated fatty acid (PUFA) metabolism:* PUFA metabolism was significantly altered in the NIST smokers’ vs. non-smokers’ urine, the NNK-treated cells vs. control cells, and the in cancerous vs. noncancerous oral tissue included, and was also described as significant in both literature reviews. PUFAs are a class of fatty acids that are heavily involved in cell signaling and immune system modulation. In the context of cancer, PUFAs are involved in the management of oxidative stress and the control of apoptotic signaling [61]. Our data suggest that oral cells may be altering PUFA metabolism to have a survival advantage during tumorigenesis, and smoking tobacco may be assisting in this process.

*Vitamin metabolism:* Alterations in vitamin metabolism was the third category of metabolic perturbations that was shared across all investigations, and many play a role as cofactors in the other super pathways. Vitamin A metabolism was described as being impacted by tobacco use in the two literature reviews (as seen by changes in β-carotene) as well as the NNK-treated vs. untreated cells. Vitamin A has been shown to have anti-carcinogenic effects in the body due to its role in regulating proliferation, differentiation, apoptosis, and antioxidant responses [62,63]. Additionally, vitamin B metabolism was described as being impacted by tobacco use in the literature review on smoking and metabolism, as well as in the NIST smoker vs. non-smoker samples, and in CAL27 cells treated with NNK. The effects of B vitamins on biochemical reactions is highly diverse as they play a role in many different reactions and processes including anabolic/catabolic reactions, oxidation-reduction reactions, regulation of proliferation, and even regulation of immune function [64]. Multiple B vitamins have been linked processes thought to play a role in carcinogenesis. Deficiencies in thiamine metabolism have been shown to decrease ATP production and increase production of reactive oxygen species that are known to have carcinogenic effects. Folate (vitamin B9) and vitamin B12 deficiencies have been shown to cause deficiencies in glutathione and increase oxidative stress, which could also play a role in carcinogenesis [64]. Lastly, the comparison of cancerous vs. noncancerous oral tissue was the only study in our investigation to show alterations in vitamin K metabolism. Vitamin K subspecies have been shown to control numerous processes related to cancer growth and development, including control of cell cycle/proliferation/apoptosis, modulation of inflammation, and control of reactive oxygen species [64]. Our results indicate that vitamin metabolism is implicated in both smoking tobacco and oral cancer. Additionally, it is known that cigarette smoke has profound effects on the gut microbiome, intestinal permeability/irrigation, and mucosal immunity [65]. Given that the microbiome is known to play a significant role in the metabolism and/or synthesis of vitamins, particularly K vitamins and B vitamins such as biotin, cobalamin, folates, nicotinic acid, pantothenic acid, pyridoxine, riboflavin, and thiamine [66]. This provides a potential mechanism for why we and others have seen changes in vitamin forms due to tobacco use. Given the role of vitamins in many endogenous metabolic pathways, this effect may also contribute to perturbations seen in other metabolic pathways. Additionally, altered microbial activity and intestinal integrity may compromise transport/absorption mechanisms for various classes of nutrients, providing another potential mechanism for metabolic perturbations seen in tobacco use.

*Steroid metabolism* Steroid metabolism was described as impacted by tobacco use in the first literature review, the NIST smokers’ vs. non-smokers’ urine experiment, and the NNK-treated vs. untreated cells. Steroids, particularly sex steroids, have primarily been studied in breast and prostate cancer where they have been shown to regulate cell proliferation and immune system activity. Beyond this, steroids are known regulators of metabolic activity and affect energy metabolism, adipogenesis, and insulin sensitivity [67]. Our investigations show that smoking tobacco or tobacco-derived carcinogens primarily target steroid metabolism, and may be a mechanism by which smoking leads to the other metabolic changes that are seen in cancer.

*Nucleotide metabolism* Nucleotide metabolism was altered in NIST smokers’ vs. non-smokers’ urine, the TMA experiment, and the CAL27 experiment. Nucleotide metabolism has been shown to play a role in maintenance of genomic stability, cellular senescence, and facilitating the transformation of normal cells to malignant cells [68]. Our investigations suggest that these alterations may happen following exposure to tobacco smoke/tobacco carcinogens, and persists following oral cancer development. Our literature review did not reveal literature on the role of nucleotide metabolism in tobacco use and oral cancer. Nucleotide metabolism may have undiscovered diagnostic or therapeutic utility in this area, indicating the potential benefit to develop therapeutics (drugs, nutrients, etc.) to target additional steps on this pathway. Additionally, simultaneously targeting multiple steps in this pathway through combination therapies may be particularly beneficial. Other metabolic disturbances that were described in the literature and our experiments, such as one carbon metabolism and folate metabolism, are closely linked to nucleotide metabolism, adding to the validity of this finding. 

## 5. Conclusions

The metabolic pathways that were altered between smokers vs. non-smokers, NNK-treated vs. untreated oral cells, and between cancerous oral tissue vs. noncancerous oral tissue may provide mechanistic insights for how smoking leads to oral cancer development. Alterations in amino acid metabolism, carbohydrate metabolism and oxidative phosphorylation, fatty acid oxidation, PUFA metabolism, and vitamin metabolism were shared across the literature reviews and the in vivo and in vitro metabolomics experiments, supporting the conclusion that these metabolic processes are most heavily implicated in tobacco-related oral cancer development. These pathways are heavily involved in fulfilling the energy demands of the cell, and suggest that smoking may lead to a rewiring of the energy-producing/consuming pathways to facilitate oncogenesis. Nucleotide metabolism, while not highlighted in the literature reviews, was found to play a significant role in differentiating smokers and non-smokers as well as health and malignant oral tissues in our metabolomics experiments. Using quantitative targeted methods, it may be possible to validate metabolites in these pathways as predictive markers for smokers at a higher risk of oral cancer in comparison to other tobacco users in future, controlled studies investigating smokers/nonsmokers who do or do not develop oral cancer. This study provides a foundation for potential pathways or specific metabolites that could serve as these biomarkers, although these future studies are needed for confirmation. Furthermore, nutritional or pharmacological therapeutics targeting these pathways may provide a strategy for preventing or treating oral cancers that develop due to tobacco use. Nutrients and nutraceuticals have been shown to have anticancer effects by targeting metabolism in other cancers [69], therefore, diet and lifestyle factors may have potential in targeting these metabolic alterations–in combination with drugs–to treat or prevent oral cancers. More research needs to be done in the future to validate the role of these metabolic perturbations in response to smoking and/or oral cancer development.

*Limitations* One limitation of our investigation is that the samples from NIST were pooled urine samples, which do not allow for the assessment of metabolic individuality. However, this can be viewed as advantageous as it diminishes the variability caused by inter-individual differences. Additionally, the TMA format contains small amounts of biological tissues, limiting the number of peaks that can be detected, and subsequently, the number of metabolites that can be identified/annotated. Also, while NNK is one of the most well-studied carcinogens in tobacco, there are many other carcinogenic compounds found in tobacco/tobacco smoke that are likely to contribute to the overall metabolic effects of tobacco. Additional studies with other tobacco carcinogens should be performed to see if similar pathways are affected. Lastly, because this was a discovery-focused study, we did not correct for multiple comparisons, as these studies were not powered for a specific hypothesis [70,71,72]. Future targeted studies with larger samples sizes, particularly for NIST smoker/nonsmoker and NNK-treated cells, are needed to fully confirm the role of these metabolites in tobacco use and oral cancer.

## Figures and Tables

**Table 1:** Literature review summary for the effects of smoking tobacco on host metabolism.

Author	Reference	Change in Compound(s) from Smoking	Sample Type	Sample Size
Gu et al.	[19]	↑ 2-, 4-hydroxylation of estrogen ↓ total parent estrogen and estrogen metabolites	Urine	603
Aug et al.	[20]	↑ ADP, α-ketoglutarate, gln, creatine, hypoxanthine, aconitic acid ↓ phospholipids, inorganic phosphate, spermidine	Primary Cells	*-*
Ghasemi et al.	[21]	↑ NO and NOx levels	Serum	333
Hsu et al.	[22]	↓ glutamate, glycerophospholipids, and cAMP	Serum	105
Nelson et al.	[23]	↑ biogenic amines ↓ dipeptides	Mid-vaginal swabs	36
Windham et al.	[24]	↑ FSH	Urine	403
Lokki et al.	[25]	↓ 25-hydroxyvitamin D	Serum	359
Zhao et al.	[26]	↑ testosterone in men but not in women	Serum	19,406
He et al.	[27]	↑ myristic acid and 3β-hydroxy-5-cholestenoic acid ↓ 7Z, 10Z-hexadecadienoic acid	Serum	70
Ulvik et al.	[28]	↓ folate, riboflavin, pyridoxal 5’phosphate (PLP)	Serum	6775
Zappacosta et al.	[29]	↑ glutathione	Saliva	40
Van der Plas et al.	[30]	↑ 11-dehydrothromboxane B2 levels	Urine	5087
Nadruz et al.	[31]	↑ N-terminal pro-brain natriuretic peptide (NT-proBNP) and high-sensitivity Troponin T (hs-TnT)	Blood	9649
Pfeiffer et al.	[32]	↑ total homocysteine (tHcy) ↓vitamin C, PLP, 4-pyridoxic acid, serum folate, and RBC folate	Blood	8944
Schectman et al.	[33]	↓ serum vitamin C	Serum	11,592
Tiboni et al.	[34]	↓ β-carotene	Plasma and follicular fluid	60
Tuenter et al.	[35]	↑ homocysteine ↓ folate and vitamin B12	Multiple	37,822
Need et al.	[36]	↓ calcium absorption rate, vitamin D metabolites, and PTH.	Blood and Urine	405
Cichosz et al.	[37]	↑ triglycerides and LDL ↓ HDL, oral glucose tolerance test (OGTT) scores	Blood	12,460
Grøndahl et al.	[38]	↑ fasting glucagon ↓ post meal glucose	Blood	23
Keser et al.	[39]	↑NO	Brain Tissue	58
Ko et al.	[40]	↑ calcium, phosphorous, and deoxypyridinoline	Urine and Serum	60
Carnevale et al.	[41]	↑ oxidative stress markers ↓ nitric oxide bioavailability and vitamin E	Blood	40
Özdemir et al.	[42]	↑ iNOS expression	Gingival tissue and gingival crevicular fluid	41

ADP, adenosine diphosphate; cAMP, cyclic adenosine monophosphate; FSH, follicle stimulating hormone; iNOS, inducible nitric oxide synthase; NO, nitric oxide; NOx, nitric oxide metabolites; PLP, pyridoxal 5′ phosphate; PTH, parathyroid hormone; RBC, red blood cell. A symbol of ↑ indicates an increase in smokers, while a ↓ indicates a decrease in smokers.

**Table 2:** Literature review summary of the metabolic features of oral cancer.

Author	Reference	Metabolic Alterations Due to Cancer Status	Population Size	Sample Type
Richie et al.	[43]	↓ iron and glutathione	200	Serum
Tiziani et al.	[44]	↑ sarcosine, dimethylglycine, betaine, choline, asparagine, ornithine, phenylalanine, glucose, acetoacetate, acetone, and 3-hydroxybutyrate ↓ levels of creatinine, creatine, gly, ser, pyruvate, ala, lactate, ile, leu, lys, thr, tyr, val, gln, pro, and citrate	25	Serum
Wei et al.	[45]	↑ n-eicosanoic acid and lactic acid ↓ γ-aminobutyric acid, phe, and val	103	Saliva
Sugimoto et al.	[46]	↑ ala, leu, Ile, his, val, trp, glu, thr, taurine, and carnitine	215	Saliva
Xie et al.	[47]	↓ cys and tyr	103	Urine
Umashree et al.	[48]	↑ nitrate and nitrite	50	Saliva
Yuvaraj et al.	[49]	↑ porphyrin	123	Saliva
Wang et al.	[50]	↓ phe and leu	90	Saliva
Bhat et al.	[51]	↑ pyruvic acid	50	Saliva and serum
Ishikawa et al.	[52]	↑ glutaminolysis, lactate, kynurenine, SAM, and pipecolate ↓ glycolysis intermediates	68	Saliva
Ishikawa et al.	[53]	↑ N,N-dimethylglycine, isopropanolamine, cystine, trimethylamine N-oxide, guanosine, hypotaurine, SAM, and pipecolate	66	Saliva
Lohavanichbutr et al.	[54]	alterations in glu, gln, gly, ser, thr, arg, pro, ala, asp metabolism and TCA cycle metabolism	100	Saliva
Chan et al.	[55]	↓ ubiquinone and β-carotene	194	Serum
Mukhopadhyay et al.	[56]	multiple alterations in central carbon metabolism	40	Oral Tissue

A symbol of ↑ indicates an increase in oral cancer, while a ↓ indicates a decrease in oral cancer.

**Table 3:** Significantly perturbed metabolic pathways that differentiated NIST smokers’ and non-smokers’ reference urine materials.

Pathway Number	Pathway Name	Combined *p*-Value
P1	Tyrosine metabolism	0.00044
P2	Butanoate metabolism	0.00046
P3	C21-steroid hormone biosynthesis and metabolism	0.00645
P4	Valine, leucine and isoleucine degradation	0.00673
P5	Vitamin B1 (thiamin) metabolism	0.01029
P6	Tryptophan metabolism	0.01565
P7	Drug metabolism-cytochrome P450	0.01626
P8	Propanoate metabolism	0.0254
P9	Glycosphingolipid metabolism	0.03312
P10	Androgen and estrogen biosynthesis and metabolism	0.03626
P11	Linoleate metabolism	0.0402
P12	Sialic acid metabolism	0.04572
P13	Biopterin metabolism	0.04786
P14	Pyrimidine metabolism	0.04879
P15	Galactose metabolism	0.0494

**Table 4:** Top 50 significantly altered metabolites between tobacco smokers’ and non-smokers’ NIST reference urine.

Peak	Ontology	Compound Name	*p*-Value	Fold Change (Smoker/ Non-Smoker)	VIP
0.63_147.0765 *m*/*z*	OL1	Glutamine	2.17 × 10^−5^	−1.7	1.2
0.64_117.0538 *n*	OL1	Guanidineacetic Acid	2.17 × 10^−5^	−1.3	1.1
0.64_75.0684 *n*	OL1	Trimethylamine Oxide	2.17 × 10^−5^	−1.2	1.2
0.71_157.0608 *m*/*z*	OL1	Formiminoglutamic Acid	2.17 × 10^−5^	1.2	1.1
0.71_161.0688 *n*	OL1	2-Aminoadipic Acid	2.17 × 10^−5^	−1.7	1.3
0.71_203.1502 *m*/*z*	OL1	N,N-Dimethyl-Arginine	2.17 × 10^−5^	1.2	1.1
0.73_163.0601 *m*/*z*	OL1	Mannose	2.17 × 10^−5^	−3.4	1.3
0.82_115.0390 *m*/*z*	OL1	Xylose	2.17 × 10^−5^	−1.8	1.3
0.82_174.0641 *n*	OL1	N-Acetylasparagine	2.17 × 10^−5^	1.2	1.2
0.82_180.0867 *m*/*z*	OL1	D-(+)-Glucosamine	2.17 × 10^−5^	−2.2	1.3
1.02_112.0273 *n*	OL1	Uracil	2.17 × 10^−5^	−1.6	1.2
1.04_147.0532 *n*	OL1	Threo-3-Methylaspartate	2.17 × 10^−5^	1.4	1.3
1.10_134.0580 *n*	OL1	Deoxyribose	2.17 × 10^−5^	−1.6	1.3
1.10_193.0972 *m*/*z*	OL1	Trans-3’-Hydroxycotinine	2.17 × 10^−5^	33.3	1.3
1.11_139.0026 *m*/*z*	OL1	Aconitic Acid	2.17 × 10^−5^	−1.3	1.2
1.18_262.1283 *m*/*z*	OL1	Succinylcarnitine	2.17 × 10^−5^	−1.3	1.2
1.20_167.0219 *n*	OL1	Quinolinate	2.17 × 10^−5^	−1.3	1.2
1.26_163.1230 *m*/*z*	OL1	Nicotine	2.17 × 10^−5^	45.2	1.3
1.48_153.0408 *m*/*z*	OL1	Xanthine	2.17 × 10^−5^	−1.6	1.3
1.60_179.1179 *m*/*z*	OL1	(1′S,2′S)-Nicotine 1′-Oxide	2.17 × 10^−5^	37.8	1.3
1.70_176.0322 *n*	OL1	1,2,3-Propanetricarboxylic	2.17 × 10^−5^	3.7	1.3
1.74_153.0408 *m*/*z*	OL1	Xanthine	2.17 × 10^−5^	−12.3	1.3
1.82_178.1106 *n*	OL1	(1′S,2′S)-Nicotine 1′-Oxide	2.17 × 10^−5^	35.1	1.3
1.86_145.0496 *m*/*z*	OL1	3-Hydroxyadipic 3,6 Lactone	2.17 × 10^−5^	−2.7	1.3
1.99_177.1023 *m*/*z*	OL1	Cotinine	2.17 × 10^−5^	31.0	1.3
2.01_162.0529 *n*	OL1	3-Hydroxyadipic Acid	2.17 × 10^−5^	−1.4	1.3
2.37_144.0424 *n*	OL1	3-Hydroxyadipic 3,6 Lactone	2.17 × 10^−5^	−1.4	1.2
2.44_127.0391 *m*/*z*	OL1	3-Hydroxy-3-Methylglutaric Acid	2.17 × 10^−5^	−1.5	1.3
2.48_267.0966 *n*	OL1	Adenosine	2.17 × 10^−5^	1.3	1.1
2.60_183.0532 *n*	OL1	4-Pyridoxic Acid	2.17 × 10^−5^	−1.4	1.2
2.62_145.0496 *m*/*z*	OL1	2-Hydroxyadipic Acid	2.17 × 10^−5^	−1.6	1.2
2.83_269.0879 *m*/*z*	OL1	Inosine	2.17 × 10^−5^	−12.3	1.3
3.01_166.0491 *n*	OL1	7-Methylxanthine	2.17 × 10^−5^	−1.6	1.3
3.19_165.0790 *n*	OL1	Phenylalanine	2.17 × 10^−5^	1.2	1.2
3.28_166.0491 *n*	OL1	3-Methylxanthine	2.17 × 10^−5^	−2.2	1.3
3.45_151.0634 *n*	OL1	Acetaminophen	2.17 × 10^−5^	−3.6	1.3
3.49_232.1543 *m*/*z*	OL1	Isobutyryl-L-Carnitine	2.17 × 10^−5^	−1.4	1.2
3.71_153.0427 *n*	OL1	3-Hydroxyanthranilate	2.17 × 10^−5^	−2.1	1.3
3.73_221.0722 *n*	OL1	N-Acetyl-S-(3-Hydroxypropyl)Cysteine	2.17 × 10^−5^	4.6	1.3
3.84_195.0532 *n*	OL1	3-Hydroxyhippuric Acid	2.17 × 10^−5^	1.4	1.2
3.88_151.0634 *n*	OL1	Acetaminophen	2.17 × 10^−5^	−4.2	1.3
4.03_196.0597 *n*	OL1	1,3-Dimethyluric Acid	2.17 × 10^−5^	−1.5	1.3
4.03_219.1107 *n*	OL1	Pantothenate	2.17 × 10^−5^	−1.3	1.2
4.22_180.0647 *n*	OL1	3,7-Dimethylxanthine	2.17 × 10^−5^	−1.9	1.3
4.64_196.0605 *m*/*z*	OL1	4-Hydroxyhippuric Acid	2.17 × 10^−5^	−7.3	1.3
5.05_235.0878 *n*	OL1	N-Acetyl-S-(3-Hydroxypropyl-1-Methyl)-L-Cysteine	2.17 × 10^−5^	3.8	1.3
5.28_122.0368 *n*	OL1	4-Hydroxybenzaldehyde	2.17 × 10^−5^	−1.4	1.3
5.28_179.0582 *n*	OL1	Hippuric Acid	2.17 × 10^−5^	−1.4	1.2
5.92_194.0804 *n*	OL1	1,3,7-Trimethylxanthine	2.17 × 10^−5^	−1.8	1.3
7.14_193.0740 *n*	OL1	4-Methylhippuric Acid	2.17 × 10^−5^	3.6	1.3

Peaks are listed in the format of retention time_mass. An “*m*/*z*” after the mass denotes a singleton ion mass whereas an “*n*” denotes a cluster of two or more adducts with the neutral mass listed. Positive fold changes denote metabolites that were higher in smokers while negative fold changes denote metabolites that were lower in smokers.

**Table 5:** Significantly perturbed metabolic pathways that differentiated malignant and oral tissues.

Pathway Number	Pathway Name	Combined *p*-Value
P1	Propanoate metabolism	0.00848
P2	Vitamin K metabolism	0.00848
P3	Polyunsaturated fatty acid biosynthesis	0.01344
P4	Histidine metabolism	0.02389
P5	Carbon fixation	0.02778
P6	Fatty acid activation	0.03208
P7	De novo fatty acid biosynthesis	0.03863
P8	Arginine and Proline Metabolism	0.04103
P9	Pentose and Glucuronate Interconversions	0.04226
P10	Pyruvate Metabolism	0.04226
P11	Linoleate metabolism	0.04284
P12	Methionine and cysteine metabolism	0.04714
P13	TCA cycle	0.04752

**Table 6:** Top 50 significantly altered metabolites between malignant and normal oral tissues.

Peak	Ontology	Accepted Compound ID	*p*-Value	Fold Change (Malignant/Normal)	VIP
6.02_265.1041 *m*/*z*	PDa	2-Aminoadenosine	1.6 × 10^−5^	1.2	1.4
13.94_347.1822 *m*/*z*	PDa	Hymeglusin	5.6 × 10^−5^	1.3	1.2
16.97_320.2577 *m*/*z*	OL2b	Eicosapentaenoate	9.6 × 10^−5^	−2.1	2.1
10.97_307.1510 *m*/*z*	PDa	5-(Galactosylhydroxy)-L-lysine	1.6 × 10^−4^	1.1	1.4
12.37_326.0001 *n*	PDa	Tris(1-chloro-2-propyl) phosphate	2.3 × 10^−4^	1.2	1.4
0.67_148.0967 *m*/*z*	PDa	4-Hydroxy-L-isoleucine	4.5 × 10^−4^	1.2	1.2
14.90_361.2341 *m*/*z*	PDa	Leukotriene B3	6.8 × 10^−4^	1.7	1.0
15.58_391.2446 *m*/*z*	PDa	15(R)-15-Methylprostaglandin F2α	7.9 × 10^−4^	−1.3	1.6
15.30_487.3235 *m*/*z*	PDa	Glycolic acid pentaethoxylate lauryl ether	9.0 × 10^−4^	3.8	1.0
8.91_227.0675 *m*/*z*	OL2a	Azelate	1.6 × 10^−3^	−1.5	1.4
13.22_491.2608 *m*/*z*	PDa	5α-Androstan-3α,17β-diol-O-3-β-glucuronic acid	2.1 × 10^−3^	−1.1	1.4
14.67_331.1001 *m*/*z*	PDa	2’,3’-Isopropylideneinosine	2.8 × 10^−3^	1.5	0.9
9.88_293.1353 *m*/*z*	PDa	His-His	3.2 × 10^−3^	1.1	1.2
0.67_112.0504 *m*/*z*	OL2a	Cytosine	3.7 × 10^−3^	1.5	0.8
14.41_377.2655 *m*/*z*	PDa	2-Linoleoylglycerol	4.0 × 10^−3^	1.2	1.3
8.03_201.1118 *m*/*z*	OL2a	3-Hydroxydecanedioic acid	5.2 × 10^−3^	1.1	1.3
11.66_271.0935 *m*/*z*	OL2a	Monocyclohexyl phthalate	5.4 × 10^−3^	3.0	1.5
16.24_423.3071 *m*/*z*	PDa	1,2-Didecanoyl-sn-glycerol	6.6 × 10^−3^	−1.1	1.3
16.54_409.2918 *m*/*z*	PDa	Leupeptin	7.9 × 10^−3^	−1.3	1.2
11.98_333.1665 *m*/*z*	PDa	[5-Formyl-2-(2-hydroxypropan-2-yl)-4a,6-dimethyl-2,3,4,7,8,8a-hexahydrochromen-4-yl] acetate	8.0 × 10^−3^	1.1	1.2
10.41_180.1016 *m*/*z*	OL2a	Propham	1.1 × 10^−2^	13.8	1.0
15.19_373.2341 *m*/*z*	PDa	5-Heptenoic acid, 7-[(1R,4S,5S,6R)-6-[(1E,3S)-3-hydroxy-1-octen-1-yl]-2-oxabicyclo [2.2.1]hept-5-yl]-, (5Z)-	1.1 × 10^−2^	−1.2	1.3
11.35_250.1200 *n*	PDa	1,2-Benzenedicarboxylic acid, dipropyl ester	1.2 × 10^−2^	1.2	1.2
4.83_189.1231 *m*/*z*	PDa	Leu-Gly	1.3 × 10^−2^	2.2	0.9
17.00_371.1005 *m*/*z*	PDa	Cyclopentasiloxane, decamethyl-	1.4 × 10^−2^	1.2	1.1
16.44_371.1005 *m*/*z*	PDa	Cyclopentasiloxane, decamethyl-	1.5 × 10^−2^	1.5	1.1
16.61_359.2160 *m*/*z*	PDa	5,8,11,14-Tetraoxa-2-azahexadecanoic acid, 16-amino-, 1,1-dimethylethyl ester	1.5 × 10^−2^	1.5	1.3
0.70_212.0914 *m*/*z*	OL2a	Malonyl-carnitine	1.5 × 10^−2^	−1.3	1.3
16.92_423.3073 *m*/*z*	PDa	1,2-Didecanoyl-sn-glycerol	1.7 × 10^−2^	−1.1	1.2
11.19_149.0228 *m*/*z*	PDa	1,2-Benzenedicarboxylic acid	1.7 × 10^−2^	1.1	1.2
14.43_314.1874 *n*	PDa	7-Oxoabieta-8,11,13-trien-18-oic acid	1.7 × 10^−2^	1.1	0.9
0.67_134.0811 *m*/*z*	OL2a	1-Aminocyclopropanecarboxylic acid	2.0 × 10^−2^	1.3	1.2
11.53_376.2012 *m*/*z*	PDa	3-[3-(Dimethylamino)propyl]-4-hydroxy-N-[4-(4-pyridyl)phenyl]benzamide	2.1 × 10^−2^	1.1	1.3
15.81_328.2605 *n*	PDa	Monopalmitolein (9c)	2.3 × 10^−2^	−1.1	1.2
16.82_409.2918 *m*/*z*	PDa	Leupeptin	2.8 × 10^−2^	1.1	1.3
16.64_425.3020 *m*/*z*	PDa	1-Octadecyl lysophosphatidic acid	2.8 × 10^−2^	1.5	0.7
16.97_386.2844 *m*/*z*	PDa	2-Methylarachidonoyl-2’-fluoroethylamide	2.9 × 10^−2^	−2.6	0.9
13.96_317.1506 *m*/*z*	PDa	4-(4-(1H-Indol-3-yl)-3,6-dihydro-1(2H)-pyridinyl)-1H-pyrazolo [3,4-d]pyrimidine	3.0 × 10^−2^	−1.8	1.1
12.32_251.1248 *m*/*z*	OL2b	5-Methylcytosine	3.2 × 10^−2^	1.1	1.0
15.40_419.3124 *m*/*z*	OL2a	5-Dodecenoic acid	3.2 × 10^−2^	1.2	1.3
14.88_421.2552 *m*/*z*	PDa	Phe-Val-Arg	3.2 × 10^−2^	1.6	1.3
7.55_288.1543 *n*	PDa	Arg-Asn	3.7 × 10^−2^	1.1	1.4
14.72_407.2761 *m*/*z*	PDa	17,20-Dimethyl Prostaglandin F1α	3.8 × 10^−2^	1.2	1.3
14.64_274.2138 *n*	PDa	Monolaurin	3.8 × 10^−2^	−1.1	1.4
10.71_231.1025 *m*/*z*	OL2a	4-hydroxybenzophenone	4.1 × 10^−2^	−4.5	1.0
8.94_180.0636 *n*	OL2b	Galactose	4.2 × 10^−2^	1.2	0.3
18.48_384.3384 *n*	OL2b	7-Dehydrocholesterol	4.3 × 10^−2^	−2.0	1.1
9.99_376.2013 *m*/*z*	PDa	N,N-dimethyl-4-[[4-(dimethylamino)phenyl](4-nitrophenyl)methyl]aniline	4.3 × 10^−2^	1.1	1.1
16.13_274.2159 *m*/*z*	PDa	α-Pyrrolidinooctanophenone	4.8 × 10^−2^	2.2	0.9
1.06_135.0026 *m*/*z*	OL2a	Glyceraldehyde	4.8 × 10^−2^	1.2	0.8

Peaks are listed in the format of retention time_mass. An “*m*/*z*” after the mass denotes a singleton ion mass whereas an “*n*” denotes a cluster of two or more adducts with the neutral mass listed. Positive fold changes denote metabolites that were higher in malignant tissues while negative fold changes denote metabolites that were lower in malignant tissues.

**Table 7:** Significantly perturbed metabolic pathways that differentiated NNK and vehicle-treated CAL27 cells.

Pathway Number	Pathway Name	Combined *p*-Value
P1	Tryptophan metabolism	0.00068
P2	Glycosphingolipid metabolism	0.00148
P3	Carnitine shuttle	0.00225
P4	Tyrosine metabolism	0.00511
P5	Androgen and estrogen biosynthesis and metabolism	0.0069
P6	Vitamin B9 (folate) metabolism	0.00977
P7	Glycerophospholipid metabolism	0.01083
P8	Porphyrin metabolism	0.01565
P9	TCA cycle	0.01565
P10	Electron transport chain	0.01578
P11	Glycosphingolipid biosynthesis-globoseries	0.02045
P12	Glycosphingolipid biosynthesis-ganglioseries	0.02419
P13	Pyrimidine metabolism	0.02521
P14	Phosphatidylinositol phosphate metabolism	0.02873
P15	Fatty acid oxidation	0.02947
P16	Linoleate metabolism	0.02994
P17	Putative anti-Inflammatory metabolites formation from EPA	0.03024
P18	Glycolysis and Gluconeogenesis	0.03086
P19	Glutamate metabolism	0.03221
P20	Purine metabolism	0.03356
P21	C21-steroid hormone biosynthesis and metabolism	0.03387
P22	De novo fatty acid biosynthesis	0.03387
P23	Fatty acid activation	0.03521
P24	Arachidonic acid metabolism	0.03565
P25	Methionine and cysteine metabolism	0.03607
P26	Drug metabolism-cytochrome P450	0.03884
P27	Xenobiotics metabolism	0.04127
P28	Blood Group Biosynthesis	0.04192
P29	Glycosphingolipid biosynthesis-lactoseries	0.04192
P30	Glycosphingolipid biosynthesis-neolactoseries	0.04192
P31	O-Glycan biosynthesis	0.04192
P32	Vitamin A (retinol) metabolism	0.04645
P33	Hyaluronan Metabolism	0.04681
P34	Fatty Acid Metabolism	0.04753
P35	Aspartate and asparagine metabolism	0.04921

**Table 8:** Top 50 significantly altered metabolites between CAL27 cells treated with NNK or vehicle.

Peak	Ontology	Compound Name	*p*-Value	Fold Change (NNK/Vehicle)	VIP
9.55_167.0700 *m*/*z*	OL2b	4-Hydroxy-3-Methoxyphenylglycol	1.0 × 10^−3^	26.8	1.5
13.96_300.2892 *m*/*z*	PDa	Phytosphingosine	2.0 × 10^−3^	1.9	1.5
0.65_118.0862 *m*/*z*	OL2a	Betaine	2.0 × 10^−3^	1.6	1.5
18.47_791.5832 *n*	PDa	Docosahexaenoyl PAF C-16	3.4 × 10^−3^	2.0	1.5
16.04_481.3528 *n*	PDa	Lyso-PAF C-16	4.2 × 10^−3^	2.2	1.5
18.81_506.3603 *m*/*z*	PDa	1-Stearoyl-2-hydroxy-sn-glycero-3-phosphocholine	4.3 × 10^−3^	3.9	1.5
17.99_728.5830 *n*	PDa	N-Oleoyl-D-erythro-sphingosylphosphorylcholine	5.0 × 10^−3^	3.0	1.5
17.70_687.4836 *n*	PDa	PE(16:1(5Z)/16:1(5Z))	5.4 × 10^−3^	3.5	1.5
3.74_154.0497 *m*/*z*	OL2a	3-Hydroxyanthranilate	5.4 × 10^−3^	3.9	1.5
3.74_232.1541 *m*/*z*	OL1	Butanoylcarnitine	6.1 × 10^−3^	1.6	1.5
1.08_175.0480 *n*	OL1	N-Acetylaspartate	6.4 × 10^−3^	2.0	1.5
16.57_509.3842 *n*	PDa	Lyso-PAF C-18	6.8 × 10^−3^	1.9	1.5
13.29_181.1221 *m*/*z*	PDa	Methyl perillate	7.4 × 10^−3^	−1.3	1.5
0.79_157.0107 *m*/*z*	OL2a	Malic acid	8.2 × 10^−3^	1.4	1.5
19.23_771.6143 *n*	PDa	1-(1Z-Octadecenyl)-2-(9Z-octadecenoyl)-sn-glycero-3-phosphocholine	8.3 × 10^−3^	1.7	1.5
13.70_299.2820 *n*	PDa	D-erythro-C18-Sphingosine	8.7 × 10^−3^	2.2	1.5
4.44_160.0756 *m*/*z*	OL1	Indoleacetaldehyde	8.8 × 10^−3^	1.8	1.5
4.90_246.1697 *m*/*z*	OL1	Valerylcarnitine	9.7 × 10^−3^	1.9	1.5
1.42_542.0684 *m*/*z*	PDa	Cyclic adenosine diphosphate ribose	1.1 × 10^−2^	2.2	1.5
2.87_150.0549 *m*/*z*	OL2b	Pyridoxal	1.1 × 10^−2^	1.9	1.5
17.70_729.5309 *n*	PDa	1,2-Dipalmitoleoyl-sn-glycero-3-phosphocholine	1.1 × 10^−2^	2.3	1.5
15.46_310.2867 *n*	PDa	14(Z)-Eicosenoic acid	1.2 × 10^−2^	−1.2	1.5
18.31_742.5371 *m*/*z*	PDa	1,2-Dipalmitoyl-sn-glycero-3-phosphoethanolamine-N,N-dimethyl	1.2 × 10^−2^	2.1	1.5
13.60_318.2998 *m*/*z*	PDa	Phytosphingosine	1.2 × 10^−2^	1.6	1.5
17.46_674.5361 *n*	PDa	SM(d18:1/14:0)	1.2 × 10^−2^	2.1	1.5
4.44_144.0807 *m*/*z*	OL2b	Indole-3-Ethanol	1.2 × 10^−2^	1.6	1.5
9.10_242.0800 *n*	OL1	Lumichrome	1.3 × 10^−2^	2.6	1.5
3.33_148.0524 *n*	OL2b	trans-Cinnamic acid	1.3 × 10^−2^	1.6	1.5
1.78_131.0946 *n*	OL1	Isoleucine	1.3 × 10^−2^	1.6	1.5
4.13_219.1105 *n*	PDa	Pantothenic Acid	1.4 × 10^−2^	1.8	1.5
19.36_506.3603 *m*/*z*	PDa	1-Stearoyl-2-hydroxy-sn-glycero-3-phosphocholine	1.5 × 10^−2^	1.7	1.5
15.46_262.2294 *n*	PDa	Farnesyl acetone	1.5 × 10^−2^	−1.2	1.5
16.36_549.3794 *n*	PDa	1-O-Hexadecyl-2-O-(2E-butenoyl)-sn-glyceryl-3-phosphocholine	1.5 × 10^−2^	1.9	1.5
1.08_172.0401 *m*/*z*	OL2a	Methionine	1.5 × 10^−2^	2.3	1.5
18.41_730.5988 *n*	PDa	N-Stearoyl-4-sphingenyl-1-O-phosphorylcholine	1.6 × 10^−2^	2.2	1.5
15.93_480.3445 *m*/*z*	PDa	1-(1Z-Hexadecenyl)-sn-glycero-3-phosphocholine	1.7 × 10^−2^	2.9	1.5
1.42_663.1081 *n*	PDa	Nicotinamide adenine dinucleotide (NAD)	1.7 × 10^−2^	1.6	1.5
18.15_715.5152 *n*	PDa	2-Linoleoyl-1-palmitoyl-sn-glycero-3-phosphoethanolamine	1.7 × 10^−2^	1.5	1.5
0.67_212.0428 *m*/*z*	PDa	Phosphocreatine	1.8 × 10^−2^	1.8	1.5
1.60_204.0629 *m*/*z*	OL2a	Tyrosine	1.9 × 10^−2^	2.0	1.5
0.96_162.0760 *m*/*z*	OL1	2-Aminoadipic acid	1.9 × 10^−2^	3.4	1.5
2.19_218.1385 *m*/*z*	OL1	Propanoylcarnitine	1.9 × 10^−2^	1.7	1.4
0.86_426.9911 *m*/*z*	PDa	Uridine 5’-diphosphate	2.0 × 10^−2^	1.6	1.4
18.15_504.3444 *m*/*z*	PDa	1-(9Z-Octadecenoyl)-sn-glycero-3-phosphocholine	2.1 × 10^−2^	2.0	1.5
16.06_327.2289 *m*/*z*	OL2a	arachidonic acid	2.2 × 10^−2^	1.3	1.4
4.44_146.0600 *m*/*z*	OL2b	Indole-3-aldehyde	2.2 × 10^−2^	1.6	1.5
13.14_224.1410 *n*	OL2b	Methyl jasmonate	2.2 × 10^−2^	−1.2	1.5
4.44_227.0788 *m*/*z*	OL2a	Tryptophan	2.3 × 10^−2^	1.6	1.4
4.44_187.0632 *n*	OL2b	Indoleacrylic acid	2.4 × 10^−2^	1.5	1.4
17.51_677.4994 *n*	PDa	1,2-Ditetradecanoyl-sn-glycero-3-phosphocholine	2.4 × 10^−2^	2.1	1.5

Peaks are listed in the format of retention time_mass. An “*m*/*z*” after the mass denotes a singleton ion mass whereas an “*n*” denotes a cluster of two or more adducts with the neutral mass listed. Positive fold changes denote metabolites that were higher in NNK-treated cells while negative fold changes denote metabolites that were lower in NNK-treated cells.

**Table 9:** Summary of common metabolic alterations between literature reviews and experiments.

Pathway Name	Smoking Literature Review	Oral Cancer Literature Review	NIST Smokers vs. Non-Smokers	TMA Malignant vs. Normal	CAL27 NNK vs. Vehicle
**Super-Pathway: Amino Acid Metabolism**	X	X	X	X	X
Tryptophan metabolism			X		X
Tyrosine metabolism			X		X
Glutamate metabolism					X
Aspartate and asparagine metabolism					X
Methionine and cysteine metabolism				X	X
Alanine and Aspartate Metabolism					
Valine, leucine and isoleucine degradation			X		
Arginine and Proline Metabolism				X	
Histidine metabolism				X	
**Super-pathway: Carbohydrate Metabolism and Oxidative Phosphorylation**	X	X	X	X	X
Glycolysis and Gluconeogenesis					X
Galactose metabolism			X		
Sialic acid metabolism			X		
TCA cycle				X	X
Pyruvate Metabolism				X	
Electron transport chain					X
**Super-pathway: Vitamin Metabolism**	X	X	X	X	X
Vitamin B9 (folate) metabolism					X
Vitamin A (retinol) metabolism					X
Vitamin B1 (thiamin) metabolism			X		
Vitamin K metabolism				X	
Vitamin B6 (pyridoxine) metabolism					X
**Super-Pathway: Fatty Acid Metabolism**	X	X	X	X	X
Carnitine shuttle			X		X
Fatty acid oxidation			X		X
De novo fatty acid biosynthesis				X	X
Fatty acid activation				X	X
**Super-pathway: Polyunsaturated Fatty Acid Metabolism**	X	X	X	X	X
Linoleate metabolism			X	X	X
Putative anti-Inflammatory metabolites formation from EPA					X
Arachidonic acid metabolism					X
Polyunsaturated fatty acid biosynthesis				X	
**Super-pathway: Steroid Metabolism**	X		X		X
Androgen and estrogen biosynthesis and metabolism			X		X
C21-steroid hormone biosynthesis and metabolism			X		X
**Super-pathway: Nucleotide Metabolism**			X	X	X
Pyrimidine metabolism			X		X
Purine metabolism					X
Pentose and Glucuronate Interconversions				X	

An “X” denotes that the pathway was identified as significant in the corresponding study.

## Data Availability

Data is available upon request.

## Supplementary Materials

The following supporting information can be downloaded at: https://www.mdpi.com/article/10.3390/ijerph191610261/s1, Table S1: Significantly altered metabolites (*p* < 0.05) between tobacco smokers’ and non-smokers’ NIST reference urine; Table S2: Significantly altered metabolites (*p* < 0.05) between CAL27 cells treated with NNK or vehicle.
